# Supplementary material for: A process evaluation of an eHealth intervention to strengthen the circle of tuberculosis care in Shigatse, Tibet, China
Source: PLOS Glob Public Health. 2025 Dec 23;5(12):e0005717. doi: 10.1371/journal.pgph.0005717 (PMC12725529; doi:10.1371/journal.pgph.0005717)
Supplement: S1 Checklist — (DOCX) [file pgph.0005717.s001.docx]

## S1 Checklist. Consolidated criteria for reporting qualitative studies (COREQ): 32-item checklist for each study

Developed from:

Tong A, Sainsbury P, Craig J. Consolidated criteria for reporting qualitative research (COREQ): a 32-item checklist for interviews and focus groups. *International Journal for Quality in Health Care*. 2007. Volume 19, Number 6: pp. 349 – 357

| No. Item | Guide questions/description | Details |
| --- | --- | --- |
| 1. Inter viewer/facilitator | Which author/s conducted the interview or focus group? | Zhitong Zhang  Tingting Yin  Bei Zhang  Yinlong Li  Qiuyu Pan |
| 2. Credentials | What were the researcher’s credentials? E.g. PhD, MD | ZZ, TY, BZ: MD, MPH  YL, QP: MD, MPH, PhD |
| 3. Occupation | What was their occupation at the time of the study? | ZZ, TY, BZ, YL, QP: Research coordinator |
| 4. Gender | Was the researcher male or female? | ZZ, BZ, YL: Male  TY, QP: Female |
| 5. Experience and training | What experience or training did the researcher have? | ZZ, TY, BZ, YL, QP: Qualitative research training, qualitative research field experience |
| 6. Relationship established | Was a relationship established prior to study commencement? | No |
| 7. Participant knowledge of the interviewer | What did the participants know about the researcher? e.g. personal goals, reasons for doing the research | None |
| 8. Interviewer characteristics | What characteristics were reported about the inter viewer/facilitator? e.g. Bias, assumptions, reasons and interests in the research topic | Primary interviewers were Chinese speaking male and female researchers with a biomedical science and public health background, between the ages of 25-35 and were of similar age, education and linguistic group to participants |
| 9. Methodological orientation and Theory | What methodological orientation was stated to underpin the study? e.g., grounded theory, discourse analysis, ethnography, phenomenology, content analysis | Sections:  Process evaluation framework  Selection of participants, data collection, and processing |
| 10. Sampling | How were participants selected? e.g., purposive, convenience, consecutive, snowball | Section: Selection of participants, data collection, and processing |
| 11. Method of approach | How were participants approached? e.g., face-to-face, telephone, mail, email | Section: Selection of participants, data collection, and processing |
| 12. Sample size | How many participants were in the study? | Section: Selection of participants, data collection, and processing |
| 13. Non-participation | How many people refused to participate or dropped out? Reasons? | All approached participants agreed to participate and no participants dropped out. |
| 14. Setting of data collection | Where was the data collected? e.g., home, clinic, workplace | Private area of home or clinics (Section: Inner setting) |
| 15. Presence of non-participants | Was anyone else present besides the participants and researchers? | No |
| 16. Description of sample | What are the important characteristics of the sample? e.g., demographic data, date | Section: Results |
| 17. Interview guide | Were questions, prompts, guides provided by the authors? Was it pilot tested? | Yes, and pilot testing was done |
| 18. Repeat interviews | Were repeat inter views carried out? If yes, how many? | No |
| 19. Audio/visual recording | Did the research use audio or visual recording to collect the data? | Audio Recording |
| 20. Field notes | Were ﬁeld notes made during and/or after the interview or focus group? | Field notes were made during interview, Memo was made after interviews |
| 21. Duration | What was the duration of the inter views or focus group? | 30-45 mins |
| 22. Data saturation | Was data saturation discussed? | Reported in Methods section: Interviews were stopped when no new concepts emerged in the final three interviews for patients and providers respectively, indicating thematic saturation had been reached based on our operational criterion. |
| 23. Transcripts returned | Were transcripts returned to participants for comment and/or correction? | No |
| 24. Number of data coders | How many data coders coded the data? | 2 |
| 25. Description of the coding tree | Did authors provide a description of the coding tree? | S2 File. (Supplementary files) |
| 26. Derivation of themes | Were themes identiﬁed in advance or derived from the data? | Section: Results |
| 27. Software | What software, if applicable, was used to manage the data? | NVivo 12 |
| 28. Participant checking | Did participants provide feedback on the ﬁndings? | No |
| 29. Quotations presented | Were participant quotations presented to illustrate the themes/ﬁndings? Was each quotation identiﬁed? e.g. participant number | Yes, each quotation was identified by a participant number |
| 30. Data and ﬁndings consistent | Was there consistency between the data presented and the ﬁndings? | Yes |
| 31. Clarity of major themes | Were major themes clearly presented in the ﬁndings? | Section: Results |
| 32. Clarity of minor themes | Is there a description of diverse cases or discussion of minor themes? | Section: Results |
