# Supplementary figures and images for: A process evaluation of an eHealth intervention to strengthen the circle of tuberculosis care in Shigatse, Tibet, China
Source: PLOS Glob Public Health. 2025 Dec 23;5(12):e0005717. doi: 10.1371/journal.pgph.0005717 (PMC12725529; doi:10.1371/journal.pgph.0005717)

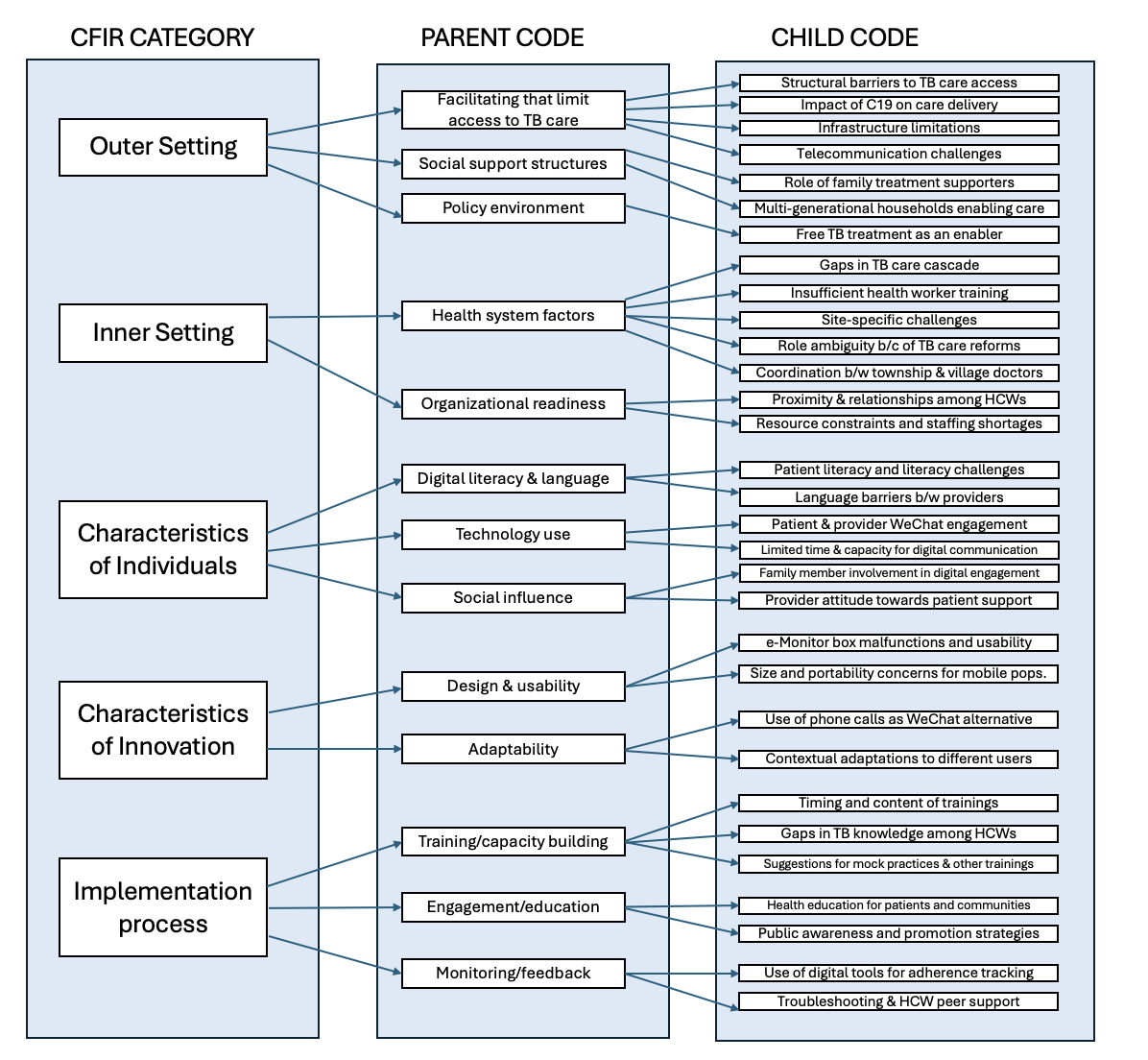

Supplement: S1 Fig — (TIF) [file pgph.0005717.s002.tif]
