# Supplementary material for: A process evaluation of an eHealth intervention to strengthen the circle of tuberculosis care in Shigatse, Tibet, China
Source: PLOS Glob Public Health. 2025 Dec 23;5(12):e0005717. doi: 10.1371/journal.pgph.0005717 (PMC12725529; doi:10.1371/journal.pgph.0005717)
Supplement: Attachment — Submitted filename: PGPH-D-25-00906-comments.pdf [file pgph.0005717.s003.pdf]

PLOS Global Public Health

A process evaluation of an eHealth intervention to strengthen the circle of tuberculosis care in Shigatse, Tibet, China

--Manuscript Draft--

**Manuscript Number:**	PGPH-D-25-00906
**Article Type:**	Research Article
**Full Title:**	A process evaluation of an eHealth intervention to strengthen the circle of tuberculosis care in Shigatse, Tibet, China
**Short Title:**	eHealth for better TB care: lessons from Shigatse, Tibet
**Corresponding Author:**	Xiaolin Wei, MD, PhD<br>University of Toronto<br>Toronto, CANADA
**Order of Authors:**	Victoria Haldane
	Zhitong Zhang
	Tingting Yin
	Bei Zhang
	Yinlong Li
	Qiuyu Pan
	Katie N. Dainty
	Elizabeth Rea
	Pande Pasang
	Jun Hu
	Xiaolin Wei, MD, PhD
**Keywords:**	Tuberculosis; e-Health; Medication adherence; Implementation science; process evaluation; Qualitative research
**Abstract:**	Background<br><br>Tuberculosis (TB) is an ongoing global health threat that has been exacerbated by the COVID-19 pandemic. People with TB need comprehensive medical and social supports to ensure they can maintain and complete TB treatment. TB programs globally have turned to eHealth to bridge gaps in access and strengthen the circle of care around people with TB.<br><br>Methods<br><br>This study evaluates the implementation of an intervention aimed at improving TB care in Shigatse, using the CFIR framework to identify factors influencing its success. The intervention included the use of e-Monitor boxes and WeChat for patient engagement. Data were collected through interviews with patients, treatment supporters, and health workers.<br><br>Results<br><br>Key challenges identified included inadequate infrastructure, digital literacy barriers, and unclear roles due to recent TB service delivery reforms. Enablers included strong social structures, proximity to village doctors, and government support for free TB treatment. Results showed that while older patients faced difficulties with digital tools, younger family members often assisted, enhancing engagement. Health workers' training and the timing of training sessions were critical to the intervention's success.<br><br>Conclusions

Powered by Editorial Manager® and ProduXion Manager® from Aries Systems Corporation

	The study concludes that despite challenges, the intervention was generally well-received and effective, with recommendations for ongoing training and adaptation to local contexts to ensure sustainability and broader TB awareness.
**Additional Information:**	
**Question**	**Response**
**Financial Disclosure**<br><br>Enter a financial disclosure statement that describes the sources of funding for the work included in this submission and the role the funder(s) played. This includes grants and any commercial funding of the work or authors.<br><br>This statement will be typeset if the manuscript is accepted for publication.<br><br>*Please review the submission guidelines and the instructions link below for detailed requirements and guidance.*	The trial is funded by TB REACH, a special initiative of Stop TB Partnership. TB REACH is supported by Global Affairs Canada.
**Competing Interests**<br><br>On behalf of all authors, disclose any competing interests that could be perceived to bias this work.<br><br>This statement will be typeset if the manuscript is accepted for publication.<br><br>*Please review the instructions link below and PLOS Global Public Health's competing interests policy to determine what information must be disclosed at submission.*	The authors declare that they have no competing interests.
**Data Availability**<br><br>Before publication, Authors are required to make fully available and without restriction all data underlying their findings. Please see our PLOS Data Policy page for detailed information on	The datasets during and/or analysed during the current study available from the corresponding author on reasonable request.

this policy.<br><br>A **Data Availability Statement**, detailing where the data can be accessed, is required at first submission. Insert your Data Availability Statement in the box below.<br><br>Please see the data reporting section of our submission guidelines for instructions on what you need to include in your Data Availability Statement.<br><br>This statement will be typeset if the manuscript is accepted for publication.<br><br>*PLOS allows rare exemptions to address legal and ethical concerns. If you have legal or ethical restrictions, please use the box below to detail these in full sentences for the Journal team to consider.*	

Powered by Editorial Manager® and ProduXion Manager® from Aries Systems Corporation

Manuscript

Click here to access/download;Manuscript;Tibet_Process eval_PLOS GPH.docx

Title: A process evaluation of an eHealth intervention to strengthen the circle of tuberculosis care in Shigatse, Tibet, China

Authors: Victoria Haldane[1], Zhitong Zhang[2], Tingting Yin[3], Bei Zhang[3], Yinlong Li[4], Qiuyu Pan[5], Katie N. Dainty[1], Elizabeth Rea[2], Pande Pasang[6], Jun Hu,[6,7] Xiaolin Wei[2]

1. Institute of Health Policy, Management and Evaluation, University of Toronto, 155 College St. Toronto, Ontario, Canada, M5T 3M6
2. Dalla Lana School of Public Health, University of Toronto, 155 College St. Toronto, Ontario, Canada, M5T 3M7
3. Weifang Medical College, Weifang, Shandong, China
4. Jining Medical University, Jining, Shandong, China
5. North Sichuan Medical College, Nanchong, Sichuan, China
6. Shigatse Centre for Disease Control and Prevention, 7 Keji Road, Samzhubze District, Shigatse, Tibet, China
7. Shandong University of Traditional Chinese Medicine, Jinan, 250355, China

Corresponding Author:

Dr. Xiaolin Wei, PhD

582-155 College St

Toronto ON, CANADA

M5T 3M7

416 978 2020

xiaolin.wei@utoronto.ca

Main text word count: 5506

Abstract

Background: Tuberculosis (TB) is an ongoing global health threat that has been exacerbated by the COVID-19 pandemic. People with TB need comprehensive medical and social supports to ensure they can maintain and complete TB treatment. TB programs globally have turned to eHealth to bridge gaps in access and strengthen the circle of care around people with TB.

Methods: This study evaluates the implementation of an intervention aimed at improving TB care in Shigatse, using the CFIR framework to identify factors influencing its success. The intervention included the use of e-Monitor boxes and WeChat for patient engagement. Data were collected through interviews with patients, treatment supporters, and health workers.

Results: Key challenges identified included inadequate infrastructure, digital literacy barriers, and unclear roles due to recent TB service delivery reforms. Enablers included strong social structures, proximity to village doctors, and government support for free TB treatment. Results showed that while older patients faced difficulties with digital tools, younger family members often assisted, enhancing engagement. Health workers' training and the timing of training sessions were critical to the intervention's success.

Conclusions: The study concludes that despite challenges, the intervention was generally well-received and effective, with recommendations for ongoing training and adaptation to local contexts to ensure sustainability and broader TB awareness.

Key words: Tuberculosis; e-Health; medication adherence; implementation science; process evaluation; qualitative research

Background

Tuberculosis (TB) remains a pressing global health threat, with disproportionate impacts in low- and middle-income countries (LMICs) [1]. Amidst ongoing health system strains, people with TB continue to face numerous barriers to accessing and maintaining treatment [2]. Adhering to lengthy treatment courses, however, is critical to reduce morbidity and mortality from TB and to prevent antimicrobial resistance, which requires lengthier and more complex treatment regimens with poorer outcomes [3, 4].

People with TB need comprehensive medical and social supports as they receive treatment. This ‘circle of care’ represents the patients’ experience of receiving care and includes health care providers and family members or ‘treatment supporters’ who rally around the person with TB and provide both medical advice and psychosocial support [5]. The strength of this circle of care depends on many factors, but ongoing access to and engagement with trained health care providers is an important component. This is particularly important considering the gold standard for TB treatment remains Directly Observed Therapy (DOTS), which by nature requires observation by a health worker [6]. Yet, for many, access to health workers is elusive, and for TB programs, DOTS is difficult to fully implement, particularly in resource-constrained and remote settings, or as COVID-19 demonstrated, when in-person visits are not possible [7, 8].

Given these challenges, TB programs globally have turned to eHealth to bridge gaps in access and strengthen the circle of care around people with TB [9, 10]. One eHealth tool increasingly used

are electronic monitoring boxes (e-monitors) that track whether TB medications are taken as directed. Studies have reported varying degrees of success in their use, highlighting the need for more research on e-monitor intervention effectiveness, cost, and implementation in resource-constrained and high-burden settings [11, 12]. This work explores one such intervention in Shigatse, Tibet Autonomous Region (Tibet), China. A randomized controlled trial was undertaken to evaluate the effectiveness of an intervention to strengthen the circle of care around people with TB using a combination of e-monitor boxes to measure treatment adherence, an app to connect people with their health care providers, and inclusion of family members as treatment supporters [13]. The trial found that using e-monitors significantly improved medication adherence and treatment success among tuberculosis patients in Tibet. Patients with e-monitors had a higher treatment success rate (94%) and better adherence compared to the control group [14].

It is important to match studies measuring effectiveness with studies to examine how the intervention works (or does not work), for whom, and why [15, 16]. Implementation science offers a complementary approach to contextualize health service intervention and is an approach to untangle and explore the rich contextual factors, and adaptations, that shape intervention implementation [5, 17, 18]. To date few studies have gathered implementation insights on TB e-monitor interventions implementation in China and none to our knowledge have explored the unique barriers and enablers present in Tibet [19-21]. Implementation experiences from Tibet can offer strategies to improve practice and policy in other resource constrained settings with a high burden of TB [22]. Guided by the Consolidated Framework for Implementation Research

(CFIR), this process evaluation draws on interviews with patients, treatment supporters and health workers to identify challenges and enablers to intervention implementation and offer ways forward for program sustainability.

Methods

This study adhered to the consolidated criteria for reporting qualitative studies (COREQ). (S1 File)

Study setting and intervention

Shigatse is a remote and sparsely populated prefecture that is characterized by high altitude and rugged terrain. These impact access to care, shape people's ability to adhere to TB treatment and the region has a TB prevalence twice the Chinese national average [23-25]. The most recent data from Shigatse found that only 72% (769/1073) of new pulmonary TB cases completed treatment, with the majority, 83% (252/304), lost to follow-up [26]. Recent TB service delivery reforms have streamlined assessment and referral processes, while strengthening TB support at the most local level – township hospitals and village doctors [27]. As part of efforts to enhance the quality of TB care in Shigatse, an eHealth intervention was developed to better connect people with TB and their health care providers.

The intervention is comprised of three key components: 1) a Bluetooth-enabled medication monitor box (e-monitor) linked to an online monitoring platform, 2) identification and

onboarding of a family treatment supporter who receives education about TB and the importance of treatment adherence, and 3) communication using WeChat (a popular messaging application platform) between people with TB, their treatment supporter, and health care providers (TB doctor and village doctor). Using these components, the intervention offered a way to strengthen the circle of care around people with TB and support them through their treatment course. The intervention being assessed aims to enhance medication adherence and improve overall treatment outcomes amongst people newly diagnosed with pulmonary TB in Shigatse [14].

Process evaluation framework

The trial and its complementary qualitative inquiry draws on the updated Consolidated Framework for Implementation Research, which encourages flexible application and offers numerous sub-constructs to help researchers probe implementation in ways that suit their research questions, innovation under study, and unique research context [17]. There is growing evidence of its use to guide study design and analysis of e-Health interventions in LMIC settings and increasing adoption of its constructs to guide process evaluations of TB interventions [28-31]. Table 1 offers the CFIR construct and its definition, as well as an overview of how we conceptualize these constructs in relation to the TB intervention being evaluated.

Table 1. CFIR framework constructs and study definitions

**Construct**	**Definition as related to our study**
Outer setting:	Relates to broader contextual factors, including local conditions and attitudes determining patient access

The setting in which the inner setting and innovation exist.	to TB care, that shape how people engage with the intervention.
Inner setting:<br>*The setting in which the innovation is implemented.*	Relates to health system organization and characteristics of implementation sites, as well as the how TB care is provided in Shigatse, Tibet.
Individuals:<br>*The roles and characteristics of individuals who engage with the innovation.*	Experiences, perspectives, and behaviours of people with TB, their treatment supporters, and health care providers that shape their experience of the intervention.
Innovation:<br>*The intervention being implemented.*	Features of the intervention that may influence its effective implementation in Shigatse, Tibet.
Implementation process:<br>*The activities and strategies used to implement the innovation.*	Description of how the TB intervention was implemented, this study both describes the process and is itself part of the process domain as it offers insights on innovation delivery.

Selection of participants, data collection, and processing

Participants included patients, their treatment supporters, and TB health workers or Centre for Disease Control staff. Patients were defined as those older than 15 years old, with presumed TB and newly confirmed pulmonary TB starting on standard 6-month short-course outpatient treatment. Family treatment supporters were those nominated family members who were participating in the intervention. Health worker participants included village doctors, township hospital doctors and nurses, county hospital doctors, as well as CDC staff working in Shigatse, Tibet. Participants were invited to the study by the research team affiliated with the randomized control trial of the intervention [13]. Purposeful recruitment was used that aimed for a balance

of gender, age, health worker roles and location across four counties representing a mix of urban and rural locations. Participants were recruited from March 1, 2019 to December 31, 2020.

Researchers conducted interviews following a topic guide tailored to different participant categories. For patients and their supporters, topics included health status, experiences with the health system, TB care, medication-taking, technology use, and engagement with the intervention (e-monitor box and WeChat). Health workers discussed their roles, TB care experiences, training, trial engagement, patient onboarding, and intervention components.
The study team included local and other researchers familiar with the study context. Interviews were held in private, comfortable locations. Interviews were conducted in Mandarin, with Tibetan interpretation as needed. Recordings were transcribed into Chinese and translated into English, with transcripts checked by a bilingual translator. More information can be found in our collaborative autoethnography describing the translation process [32].

Participant confidentiality in analysis and writing was ensured by using participant numbers and quoting participants using pseudonyms. Data was securely stored as per protocols defined by the University of Toronto Office of Research Ethics including password protection and use of secure drives.

The analysis was guided by a qualitative descriptive approach, which is often used in cross-cultural health services research given its emphasis on literal interpretation of translated narrative and limited movement 'into' the text [33-35]. Interviews were coded using framework

analysis as described by Ritchie and Lewis and guided by the CFIR constructs to categorize our data [17, 36]. NVivo 12 was used to organize the data and code transcripts. Two reviewers (VH, ZZ) reviewed the CFIR-guided coding framework based on a subset of interviews and discussed how the CFIR constructs. After discussion and agreement, the coding framework was applied to subsequent manuscripts. Interviews were stopped when it was decided that no new concepts were being heard in subsequent interviews [37].

Ethics approval and consent to participate

Ethical approval for the study was obtained from the Office of Research Ethics at the University of Toronto (Ref: 36569) and the Ethics Review Committee of the Shigatse Centre for Disease Control and Prevention (Ref: 006). Participants received information and consent forms in Chinese, translated into Tibetan if needed. Written informed consent was obtained from participants or their parents (if participants aged below 18). All participants consented to audio recording and could refuse questions or withdraw at any time.

Results

A total of 62 interviews were conducted with people with TB, their treatment supporters, and health care providers. The study was conducted in three rural counties (Gyangze, Sa'gya, and Tingri) and one urban district (Samzhubze). Interviewees were purposively sampled based on

district to ensure representation from different settings and from different categories of stakeholders who engage with the intervention (Table 2).

Table 2. Interview participants by category and study district.

**Participant characteristics**	**n=**
Patient	20
Family treatment supporter	5
Village doctor	14
Township hospital doctor/nurse	14
County hospital doctor	7
CDC staff	2
**District/county**	
Samzhubze	16
Sa'gya	17
Gyangze	13
Tingri	16

We found that patients, their supporters, and health workers were generally satisfied with the intervention components. Using the CFIR framework, we organize our results by factors in the outer and inner settings that either challenged or enabled implementation. We then describe individuals' abilities to engage with the intervention and the components themselves, especially the e-Monitor box. Additionally, we discuss challenges during implementation and factors that facilitate the process and will support ongoing delivery. Table 3 provides an overview of challenges and enablers mapped to the CFIR constructs.

Table 3. Implementation challenges and enablers presented by CFIR construct.

Construct	***Theme***

	Challenges	***Enablers***
Outer setting:	• *Factors that limit patient access to TB care*	• *Social structure of large families living together enable multiple treatment supporters to take on different roles*
Inner setting:	• *TB policy at the highest levels affect innovation implementation*<br>• *Recent TB service delivery reforms created a transition period where roles and responsibilities were unclear*	• *Proximity to village doctors and strong links between township doctors and village doctors supports innovation delivery*<br>• *Free TB treatment from the government enables retention in TB care*
Individuals and innovation:	• *Older patients face digital literacy and language barriers to using WeChat*<br>• *The e-Monitor box design is not convenient for younger patients*<br>• *Health workers have limited time to use WeChat*	• *Mobile phones enable communication in ways beyond the intervention components*<br>• *HCWs teach patients about WeChat and providing support for the e-Monitor*<br>• *e-Monitor alarm function enables timely medication taking*

Implementation process:	• *Timing of training and gaps in HCW training on innovation components shape innovation delivery*	• *The innovation can be further embedded when used as a tool for broader education on TB in the region*<br>• *Patients, treatment supporters, health workers, and community members can be leveraged to champion the intervention*

Outer setting:

The outer setting represents broad contextual factors that shape the implementation context and create (or inhibit) facilitating conditions for innovation use and engagement. Our interviews with patients and their treatment supporters revealed several factors limiting patient access to TB care, which we have described in detail elsewhere [23]. A health worker summed up the three overarching challenges to TB care in the region as: *"One, the area is highly prevalent of tuberculosis; Two, the area does not have the best healthcare environment; and three, the public has no understanding of tuberculosis,"* [T55_Township doctor_Tingri]. These challenges shape barriers identified by patients included physical access challenges, out-of-pocket costs, medication side effects, and concerns about treatment effectiveness; while enablers to TB care included knowledge or past experience with TB, integrated care models, supportive village doctors, free TB treatment, subsidies, and family and social support [23].

Inner setting:

The inner setting relates to health system factors that shape innovation use including health system organization and characteristics of implementation sites, as well as the how TB care is provided. In Shigatse, TB policies and the uncertainty in roles and responsibilities during the transition period following recent TB service delivery reforms were the main challenges reported. Despite these challenges, the role of village doctors within the health system was reported as a key enabler to intervention implementation. In addition, COVID-19 shaped the inner setting and will continue to affect the delivery of TB care in the region.

Our interviews with providers revealed how limited implementation of the Chinese National Tuberculosis Plan in Tibet has shaped the inner setting and affected innovation implementation, which has been described elsewhere [38, 39]. Health workers and CDC staff emphasized that TB programs and improving the quality of TB care must become a top policy priority in Shigatse for the intervention to succeed and be sustained over time. More broadly, one CDC Staff emphasized that efforts must be made to break down the silo-ed approach to TB service delivery from the highest levels, *"I think we need to increase awareness and promote inter-department cooperation, like [we have done for] COVID-19, find all the patients and provide proper responses…"* [T62_CDC Staff_Samzhubze]. Recent reforms have been the first step towards greater alignment with the NTP and the intervention represents a concerted effort to improve

the quality of TB care across Shigatse through more team-based TB care coordinated through township and county hospitals.

Yet, health workers and CDC staff also reported how recent TB service delivery reforms in the region created a transition period where roles and responsibilities for TB care were unclear. As one senior CDC staff explained

> *"The CDC started handing over the TB prevention responsibility to the hospitals in June 2019. After the handover, the whole TB prevention efficiency and quality dropped, because although we organized orientations at the hospitals, many details in the TB prevention process were neglected… the communication and transition between the CDC, township hospitals, as well as village clinics lacked clarity and efficacy at first due to unfamiliarity of the newly divided responsibilities. Now we are starting to get back on track again,"* [T62_CDC Staff_Samzhubze].

The learning curve associated with the reforms was also seen in the intervention. Early-on some health workers were unclear on their scope of duties and followed a more 'traditional' and hierarchical model rather than a team-based care approach with village health workers. Overall though, the role of village doctors was emphasized as a key feature of the health system that supported intervention implementation and strengthened the circle of care [38]. Their proximity and close relationships with communities, as well as their strong links with township doctors enabled village doctors to effectively deliver intervention components. As one county hospital

doctor explained, *"Explain to the patients about the advantages of E-monitor box. If the explanation isn't clear, go to the grassroot level, let village doctors in the village supervise and encourage them. The village doctors can supervise during meetings, the village doctors play a very important part,"* [T41_County hospital doctor_Sa'gya]. Supporting village doctors to safely provide high quality TB care requires ongoing training that acknowledges their crucial role in linking communities to care and that further establishes them as members of a multidisciplinary TB care team.

Individuals and innovation

The individuals category of the CFIR describes the roles and characteristics of individuals who engage with the innovation and technology-oriented factors that shape innovation use including ease of use and complexity of the technology.

Overall people were satisfied with the intervention and able to engage with the WeChat app and eMonitor box. Most health workers reported that the intervention could improve patient care, and all patients and treatment supporters reported that engaging in the intervention was beneficial. As one treatment supporter reported "*Before, it was the patient's brother that ensures that she takes the medications, after the distribution of e-Monitor box, the patient takes the medications herself,"* [T42_Family treatment supporter_Tingri]. Older patients specifically highlighted the usefulness of the alarm feature of the e-Monitor box and all patients contacted the village doctor or township doctor to successfully troubleshoot transient issues that arose.

One 64-year-old patient illustrated the ease of using the e-Monitor box by explaining that *“I can't read. [My phone] can only be used to make calls… I use the [e-Monitor] pill box every day. It can remind me to take medicine…no one [helps me] I am using it myself,”* [T34_Patient_Sa’gya).

However, participants faced various individual-level challenges and barriers in engaging with the text-based WeChat parts of the intervention, largely shaped by language, literacy, and digital literacy. These did not impact e-Monitor use but shaped how participants engaged with their circle of care.

Language and literacy challenges included limited digital literacy as well as limited written literacy and differing levels of literacy between written and spoken Mandarin and Tibetan. Many patients and treatment supporters older than 40 reported only using their phones for voice calls and not using WeChat at all. For others, language barriers prevented them from using WeChat with health workers who write in Chinese characters, as one patient explained *“I make phone calls, I will also watch videos on my phone. I don't send text messages because I cannot recognize Chinese characters. I type in Tibetan in WeChat,”* [T12_Patient_Samzhubze]. From the health worker perspective, some township hospital doctors reported language barriers between village doctors and patients that limited their use of WeChat for communication. As one township doctor explained, *“Some village doctors also don’t know how to use WeChat to communicate with patients, it is a bit more difficult. They can understand Chinese characters but can’t speak Mandarin.”* [T09_Township hospital doctor_Sa’gya]. In Shigatse, eHealth interventions need to

be flexible to ensure all literacy and language levels are able to engage with the core components of the intervention.

To overcome these barriers, participants and health workers adapted the intervention through alternative communication methods. The most common adaptation to the intervention was to use phone calls instead of WeChat, followed by house visits, to check in on patients. These adaptations still allowed for patients and treatment supporters to engage more regularly with health workers. As one village doctor described, *"WeChat is used relatively less, because patients don't have WeChat, if there's a problem, we usually go to their door for follow-up or follow-up through the phone,"* [T15_Village doctor Samzhubze]. Some patients have reported relying on family members to help them use technology [23]. For some participants, younger family members were integral in facilitating WeChat use or using WeChat on behalf of the patient. As one 56-year-old patient illustrated, *"Usually 14 people live in the house…My family members remind me, usually it will be my wife or mother reminding me. My wife is my supporter. But because we do not use WeChat, our daughter-in-law became the supporter,"* [T02_Patient_Samzhubze]. However, these social structures are shifting, and more often younger generations are migrating to urban centres for work and school. A township hospital doctor described how this can impact the intervention, *"If the WeChat [account] we 'befriended' belongs to the family members, many family members go out for work, they do not understand the patient's true condition,"* [T26_Township hospital doctor_Sa'gya]. In these cases, the intervention may need to be adapted with closer follow up by the village doctor.

Some township hospitals doctors reported that instructing village doctors and patients on how to use WeChat became a part of their role in delivering the intervention. A few, however, reported that teaching WeChat took time away from other activities. As one township doctor explained, "*Many patients don't know how to use WeChat, we can teach the patient how to use WeChat through health education and promotion…If they went to our base, we would teach the patient and the accompanying village doctor how to use WeChat one-on-one…In total [it takes] about one hour, it takes longer to set up WeChat, takes about half an hour from account registration to start of usage"* [T27_Township hospital doctor_Sa'gya].

Some groups faced challenges more difficult to overcome, including students and government workers. Younger age groups easily used WeChat, but the e-Monitor itself created a barrier to adherence given its size and visibility, as it is a bright blue box. One township doctor described specific problems faced by students, *"Students go to school in Mainland China, and because of many inconveniences, will return the pillbox, and then after the holidays, will take the pillbox back home and continue using it…It isn't convenient to carry it at school and there are problems with plugging it in at school. He takes the tuberculosis medication in secret as he does not want his classmates to know that he has tuberculosis,"* [T27_Township doctor Sa'gya]. A county hospital doctor expanded on groups who may not be able to use the e-Monitor explaining how *"Students, drivers, government employees think that the e-monitor box is too big and inconvenient to carry, so they are reluctant to use it…The project of electronic medicine box is good, but students and herdsmen will think it is too big and inconvenient to carry without carrying it,"* [T41_County hospital doctor_Sa'gya].

Implementation process

The implementation process describes the activities and strategies used to implement the innovation. The timing of training was a challenge to effective implementation and gaps in health worker training on the intervention components shaped its delivery. The implementation process, however, presents an important opportunity for broader education on TB in the region, both for health workers and the general population.

Gaps in health worker knowledge on the intervention were attributed to the time delay between training on the intervention and first patient enrolment and the need for self-directed learning to operate the e-Monitor box system. One township doctor described their situation as *"We had our first patient two months after training, we also had to discover how to operate the system by ourselves based on the operation manual. I had forgotten about the section on village doctors, and I probably did not read the village doctor section of the operation manual... I have read the training supervising manual for township hospital doctor, it is okay, I can understand the content,"* [T22_Township hospital doctor_Gyangze].

Others emphasized the need for greater detail in the training sessions and ongoing engagement with the intervention designers for refresher training as the intervention rolled out. As a county hospital doctor explained *"The training content is sufficient; but some places regarding the e-Monitor box are a bit vague and can't be understood; training materials were distributed; no*

discussions were organized after training, but we [health worker]) discuss about them during treatment," [T39_County hospital doctor_Tingri]. Health workers had ideas for improvement including holding joint training with different health workers and participating in mock scenarios. A township hospital doctor reported that *"I think, when conducting training, it would be better to make village, township hospital, and [county] hospital doctors all attend, sometimes we forget things when supervising the village doctors. It would be better to do mock practices…All project participants should participate in the training together, village doctor, township hospital doctor, country hospital doctor. Work tasks should have model case scenarios, live operation [of the e-Monitor], and actual operation process,"* [T22_Township hospital doctor_Gyangze]. While others suggested to have training twice a year to ensure health workers understood their roles and responsibilities in implementing the intervention.

Some health workers highlighted how the current training curricula could be an opportunity to address limited knowledge of TB amongst health workers in the region. Training could then introduce important concepts such as multi-drug resistant tuberculosis and new tools for diagnosis and treatment to all levels of health worker. A township hospital doctor explained that "*The orientation is somewhat lacking in lecturing relevant information on tuberculosis, such as what is tuberculosis, how to manage tuberculosis, how to diagnose suspected patients and so on…Usually training at the municipal and autonomous regions is only for county-level staff. If possible, the usual training can incorporate staff at the township level, as some knowledge is not transmitted to the township and village levels,"* [T26_Township doctor_Sa'gya]. Some village doctors similarly called for increased training on TB to be built into the orientation with one

calling for implementers *"To teach more basic knowledge such as basic vaccination and diagnosis..."* [T29_Village doctor_Sa'gya]. Others suggested that training must be done in Tibetan language to ensure all health workers have adequate knowledge of TB, particularly village doctors who are often the first point of patient contact with the health system. Participants also emphasized the role of the government in ensuring that documentation and processes are in place for this training and to support health workers more broadly. As one township hospital doctor reported, *"The government has to put a strong emphasis on this task [TB care] and publish some relevant paperwork and allow for the active screening of the disease. Since patients are less motivated to be actively seeking medication attention, medical and clinical institutions should organize more orientations for the doctors on the use of medications,"* [T26_Township doctor_Sa'gya]. Strengthening TB care in the region, and improving the quality of care delivered, requires an adequately trained health work force beginning at the most local levels with a shared understanding of TB and how to identify, refer, diagnose, and treat people presenting with the symptoms of TB.

Some health workers offered strategies to increase intervention uptake, address specific concerns, and promote intervention and TB awareness amongst patients. In Sa'gya, health workers described how some patients were initially hesitant about the e-Monitor but that health education about TB and the goal of the intervention can increase their interest in using the e-Monitor. As one township doctor explained *"Put the emphasis on health education, you should promote and educate the main function of the e-Monitor box more, the main goal of the e-Monitor box, if the patients knew about these information they'd be more willing to accept the e-*

Monitor box," [T27_Township doctor_Sa'gya]. A village doctor explained how implementation can be strengthened by engaging more deeply at the most local levels, *"From the perspective of advertisement, village health officials should promote it to patients who easily forget to take medicine. Other villagers also need to know about this project, so the project will be more acceptable if there is another patient,"* [T20_Village doctor_Samzhubze]. Another suggested that, *"It would be better for the village head to come in and persuade the patients, it is easier this way,"* [T61_Village doctor_Tingri]. These perspectives underscore the importance of engaging communities broadly in the intervention and the importance of community opinion to influence uptake and use. To this end, patients can also be champions for the intervention and to spread TB awareness, as one patient explained, *"Sometimes when I encounter patients with symptoms like mine, I will persuade them to go to the township or county hospitals to check and diagnose the disease. I persuade them for early detection and early treatment,"* [T35_Patient_Sa'gya]. Similarly, treatment supporters who had existing awareness of TB strongly supported the intervention and perceived its benefits for their family and others. To ensure intervention sustainability, awareness and engagement at all levels is key, while also addressing broader awareness of TB through better education and health worker training.

Discussion

In this process evaluation, we explore the perspectives of patients, family treatment supporters, and health workers to describe challenges and enablers to implementing an eHealth intervention to strengthen the circle of care around people with TB in Shigatse, Tibet. Using the CFIR and

UTAUT frameworks we highlight how context, individual and innovation-related factors, as well as the implementation process influence how the intervention is delivered. We also describe how adaptations were made to core intervention components in response to contextual factors. Through this analysis we discuss considerations to ensure that e-Monitor-based interventions strengthen the circle of care as they are implemented and scaled up in Shigatse and offer learnings for intervention implementation in similar high TB burden, rural and remote settings.

Implementers should anticipate adaptations of eHealth components as users find ways to create their circle of care given their unique settings and circumstances. These adaptations must be assessed to determine if they support or undermine intervention goals. For example, our study corroborates another e-Monitor study in Mainland China, which also found that older users were satisfied with e-Monitors and able to use them independently [40]. However, our study also confirms eHealth work that shows older users, particularly those living in Western China and those with lower levels of education, have accessibility and language challenges in using WeChat [41]. In Shigatse, intervention adaptations were made to ensure regular communication and a strengthened circle of care between older participants and health care providers, either through phone calls or home visits, thus achieving the goal of the patient-facing WeChat component. For younger age groups, WeChat was easily used but the e-Monitor was less convenient, particularly for mobile groups such as students. Other studies of e-Monitors in mainland China have similarly highlighted the unique needs of students in the use of e-Monitors, and globally a gap has been identified in the use of digital technology to support young people and children with TB [20, 42]. In our study, students adapted the intervention by using the e-Monitor only when they were in

Shigatse. This adaptation, however, undermines the delivery of this component as adherence cannot be monitored. Needs assessments can help implementers anticipate different use cases and to develop effective adaptations ahead of intervention implementation [40]. In addition, iterative and human-centered design methods can help implementers to understand and respond to user experiences that shape e-Monitor use during scale up [43].

Successful implementation, and indeed successful adaptation, requires timely and comprehensive training for involved health workers on all components of the intervention and how these components relate to project goals [20]. In our study, there was variability in training on the intervention particularly amongst village doctors, time delays between training and implementation, and participants called for refresher training to successfully sustain the intervention. Another TB adherence intervention involving lay health workers in Malawi, a similarly high-burden setting, reported variability in intervention training as a barrier to implementation. The authors emphasized the importance of comprehensive training for all health workers, including lay health workers, when implementing community interventions to improve TB care [44]. In addition, having a comprehensive understanding of the intervention is crucial to ensure that adaptations contribute to intervention success. Having a complete view of the intervention, their role in it, and the roles of others, allows health workers to identify implementation barriers, suggest and document actionable and effective adaptations.

Intervention training also offers an important opportunity to improve health worker knowledge of TB and its management. An adequately trained health workforce, starting at the most local

levels (e.g., village doctors in this setting) are cornerstones of global efforts to improve the quality of TB care [45, 46]. Our prior work in the region underscored low awareness of TB amongst health workers, a common challenge in remote and rural settings [38]. Across levels of care, our participants, from village doctors to county hospital doctors, highlighted their desire for greater knowledge on TB etiology, diagnosis, treatment, and risks of antimicrobial resistance. Alongside intervention training during onboarding, WeChat may offer an additional platform for health worker training and delivering TB education in rural China [47]. Although current evidence has only shown modest improvements in provider knowledge following WeChat training, more research is needed, particularly amongst health workers who have been sensitized through engagement with an intervention such as this one.

For long term success and sustainability, intervention implementation must be integrated with, and offer insights on how to achieve, broader TB goals and health policy objectives, such as increasing universal health coverage [48, 49]. Using the CFIR framework and guided by the program theory, our work found that in Shigatse, recent TB service delivery reforms provide an opportunity to scale up the intervention and align with regional goals, as well as support national efforts to reduce to reduce the burden of TB in China through greater use of eHealth and more people-centred care [27, 50]. Understanding intervention implementation offered insights on how to provide more people-centred TB care, increase access to TB health services for remote populations, raise greater awareness of TB symptoms and screening, align health workers on TB care delivery protocols, and better embed eHealth in TB health service delivery. In this way, framework and theory-guided implementation science is not only a driver of practice and health

system improvements, but also offers actionable insights for decisionmakers determining health policy. Future research should closely engage with decisionmakers to ensure implementation insights strengthen the delivery of high-quality TB care.

Strengths and limitations

This manuscript is strengthened by the perspectives of patients, treatment supporters and health workers in Shigatse, Tibet. A further strength is our use of CFIR and UTAUT frameworks to guide this process evaluation and identify challenges and enablers to intervention implementation. To our knowledge, this is the first study to explore the implementation process of an eHealth program to strengthen TB care in Shigatse. This work was further strengthened by our use of semi-structured interviews in both Tibetan and Mandarin, which enabled participants to describe their experiences in greater detail.

A key limitation of the study is desirability bias where participants may have presented a more positively framed account of their engagement with the intervention. We attempted to limit the influence of this bias by reassuring participants that the study was not related to their TB care or work performance, and that all responses would be kept confidential. The impact of this bias was likely minimal as participants were candid in sharing their perspectives, even negative ones. Although we tried to recruit a breadth of participants, given vast travel distances and limited resources, we may be missing some perspectives that could limit our interpretation of the implementation process

Conclusion

Using the CFIR framework, this qualitative process evaluation identified several challenges and enablers that shape the implementation of a multi-component eHealth intervention to strengthen the circle of TB care in Shigatse, Tibet, China. Importantly, we highlight intervention adaptations, the ways in which these meet user needs, and how some adaptations support project goals, while others can undermine them. Based on these results, we offer key takeaways for TB eHealth intervention implementation that underscore the importance of infrastructure in creating a consistent user experience, the need to anticipate and document adaptations across user groups, the role of training as a way to strengthen intervention role while capacity-building health workers, and the need for implementation science to feed into health policy objectives. This work highlights the importance of using implementation science to better understand the implementation context and the experiences of users engaging with interventions in practice, particularly in resource-constrained settings.

List of abbreviations

CFIR: Consolidated Framework for Implementation Research

COVID-19: Coronavirus disease 2019

DOT: Directory Observed Therapy

LMIC: Low- and middle-income countries

Shigatse CDC: Shigatse Centre for Disease Control

TB: Tuberculosis

Acknowledgements: The authors would like to acknowledge all members of the trial and the support teams and staff at the respective institutions for their contributions to this study. We would like to thank the participants for their cooperation and sharing, the data collectors for their efforts, and the translation team for their dedication.

References:

1. World Health Organizaton. Global Tuberculosis Report 2021. Geneva: World Health Organization, 2021.

2. Pai M, Kasaeva T, Swaminathan S. Covid-19's Devastating Effect on Tuberculosis Care - A Path to Recovery. N Engl J Med. 2022;386(16):1490-3. Epub 2022/01/06. doi: 10.1056/NEJMp2118145. PubMed PMID: 34986295.

3. Nezenega ZS, Perimal-Lewis L, Maeder AJ. Factors Influencing Patient Adherence to Tuberculosis Treatment in Ethiopia: A Literature Review. Int J Environ Res Public Health. 2020;17(15). Epub 2020/08/08. doi: 10.3390/ijerph17155626. PubMed PMID: 32759876; PubMed Central PMCID: PMCPMC7432798.

4. World Health Organizaton. Adherence to long-term therapies: evidence for action. 2003.

5. Bauer MS, Damschroder L, Hagedorn H, Smith J, Kilbourne AM. An introduction to implementation science for the non-specialist. BMC Psychol. 2015;3(1):32. Epub 2015/09/18. doi: 10.1186/s40359-015-0089-9. PubMed PMID: 26376626; PubMed Central PMCID: PMCPMC4573926.

6. Stop TB Partnership. An expanded DOTS framework for effective tuberculosis control. Int J Tuberc Lung Dis. 2002;6(5):378-88. Epub 2002/05/22. PubMed PMID: 12019913.

7. Xu B, Dong HJ, Zhao Q, Bogg L. DOTS in China - removing barriers or moving barriers? Health Policy Plan. 2006;21(5):365-72. Epub 2006/08/31. doi: 10.1093/heapol/czl019. PubMed PMID: 16940302.

8. Tadesse T, Demissie M, Berhane Y, Kebede Y, Abebe M. Long distance travelling and financial burdens discourage tuberculosis DOTs treatment initiation and compliance in Ethiopia: a qualitative study. BMC Public Health. 2013;13:424. Epub 2013/05/03. doi: 10.1186/1471-2458-13-424. PubMed PMID: 23634650; PubMed Central PMCID: PMCPMC3644232.

9. Subbaraman R, de Mondesert L, Musiimenta A, Pai M, Mayer KH, Thomas BE, et al. Digital adherence technologies for the management of tuberculosis therapy: mapping the landscape and research priorities. BMJ Global Health. 2018;3(5). doi: 10.1136/bmjgh-2018-001018.

10. Falzon D, Timimi H, Kurosinski P, Migliori GB, Van Gemert W, Denkinger C, et al. Digital health for the End TB Strategy: developing priority products and making them work. Eur Respir J. 2016;48(1):29-45. Epub 2016/05/28. doi: 10.1183/13993003.00424-2016. PubMed PMID: 27230443; PubMed Central PMCID: PMCPMC4929075.

11. Ridho A, Alfian SD, van Boven JFM, Levita J, Yalcin EA, Le L, et al. Digital Health Technologies to Improve Medication Adherence and Treatment Outcomes in Patients With Tuberculosis: Systematic Review of Randomized Controlled Trials. J Med Internet Res. 2022;24(2):e33062. Epub 2022/02/24. doi: 10.2196/33062. PubMed PMID: 35195534; PubMed Central PMCID: PMCPMC8908199.

12. Ngwatu BK, Nsengiyumva NP, Oxlade O, Mappin-Kasirer B, Nguyen NL, Jaramillo E, et al. The impact of digital health technologies on tuberculosis treatment: a systematic review. Eur Respir J. 2018;51(1). Epub 2018/01/13. doi: 10.1183/13993003.01596-2017. PubMed PMID: 29326332; PubMed Central PMCID: PMCPMC5764088.

13. Wei X, Hicks JP, Pasang P, Zhang Z, Haldane V, Liu X, et al. Protocol for a randomised controlled trial to evaluate the effectiveness of improving tuberculosis patients' treatment adherence via electronic monitors and an app versus usual care in Tibet. Trials. 2019;20(1):273. Epub 2019/05/18. doi: 10.1186/s13063-019-3364-x. PubMed PMID: 31097023; PubMed Central PMCID: PMCPMC6521492.

14. Wei X, Hicks JP, Zhang Z, Haldane V, Pasang P, Li L, et al. Effectiveness of a comprehensive package based on electronic medication monitors at improving treatment outcomes among tuberculosis patients in Tibet: a multicentre randomised controlled trial. Lancet. 2024. Epub 2024/02/04. doi: 10.1016/S0140-6736(23)02270-5. PubMed PMID: 38309280.

15. Bauer MS, Kirchner J. Implementation science: What is it and why should I care? Psychiatry Res. 2020;283:112376. Epub 2019/05/01. doi: 10.1016/j.psychres.2019.04.025. PubMed PMID: 31036287.

16. Durlak JA, DuPre EP. Implementation matters: a review of research on the influence of implementation on program outcomes and the factors affecting implementation. American Journal of Community Psychology. 2008;41(3-4):327-50. doi: 10.1007/s10464-008-9165-0.

17. Damschroder LJ, Reardon CM, Widerquist MAO, Lowery J. The updated Consolidated Framework for Implementation Research based on user feedback. Implement Sci.

2022;17(1):75. Epub 2022/10/31. doi: 10.1186/s13012-022-01245-0. PubMed PMID: 36309746; PubMed Central PMCID: PMCPMC9617234.

18. Damschroder LJ, Aron DC, Keith RE, Kirsh SR, Alexander JA, Lowery JC. Fostering implementation of health services research findings into practice: a consolidated framework for advancing implementation science. Implementation Science. 2009;4(1):50. doi: 10.1186/1748-5908-4-50.

19. Wang N, Zhang H, Zhou Y, Jiang H, Dai B, Sun M, et al. Using electronic medication monitoring to guide differential management of tuberculosis patients at the community level in China. BMC Infect Dis. 2019;19(1):844. Epub 2019/10/17. doi: 10.1186/s12879-019-4521-2. PubMed PMID: 31615433; PubMed Central PMCID: PMCPMC6794727.

20. Wang N, Shewade HD, Thekkur P, Zhang H, Yuan Y, Wang X, et al. Do electronic medication monitors improve tuberculosis treatment outcomes? Programmatic experience from China. PLoS One. 2020;15(11):e0242112. Epub 2020/11/10. doi: 10.1371/journal.pone.0242112. PubMed PMID: 33166361; PubMed Central PMCID: PMCPMC7652336.

21. Liu X, Lewis JJ, Zhang H, Lu W, Zhang S, Zheng G, et al. Effectiveness of Electronic Reminders to Improve Medication Adherence in Tuberculosis Patients: A Cluster-Randomised Trial. PLoS Med. 2015;12(9):e1001876. Epub 2015/09/16. doi: 10.1371/journal.pmed.1001876. PubMed PMID: 26372470; PubMed Central PMCID: PMCPMC4570796.

22. Cattamanchi A, Berger CA, Shete PB, Turyahabwe S, Joloba M, Moore DA, et al. Implementation science to improve the quality of tuberculosis diagnostic services in Uganda. J Clin Tuberc Other Mycobact Dis. 2020;18:100136. Epub 2019/12/28. doi:

10.1016/j.jctube.2019.100136. PubMed PMID: 31879703; PubMed Central PMCID: PMCPMC6920311.

23. Haldane V, Zhang Z, Ma Q, Yin T, Zhang B, Li Y, et al. A qualitative study of perspectives on access to tuberculosis health services in Xigaze, China. Infect Dis Poverty. 2021;10(1):120. Epub 2021/09/22. doi: 10.1186/s40249-021-00906-4. PubMed PMID: 34544492; PubMed Central PMCID: PMCPMC8451167.

24. Zhang J, Yang Y, Qiao X, Wang L, Bai J, Yangchen T, et al. Factors Influencing Medication Nonadherence to Pulmonary Tuberculosis Treatment in Tibet, China: A Qualitative Study from the Patient Perspective. Patient Prefer Adherence. 2020;14:1149-58. Epub 2020/08/09. doi: 10.2147/PPA.S252448. PubMed PMID: 32764888; PubMed Central PMCID: PMCPMC7360411.

25. Li B, Zhang X, Guo J, Wang J, Pianduo B, Wei X, et al. Prevalence of pulmonary tuberculosis in Tibet Autonomous Region, China, 2014. The International Journal of Tuberculosis and Lung Disease: The Official Journal of the International Union Against Tuberculosis and Lung Disease. 2019;23(6):735-40. doi: 10.5588/ijtld.18.0614.

26. Wei X, Hicks JP, Pasang P, Zhang Z, Haldane V, Liu X, et al. Protocol for a randomised controlled trial to evaluate the effectiveness of improving tuberculosis patients' treatment adherence via electronic monitors and an app versus usual care in Tibet. Trials. 2019;20. doi: 10.1186/s13063-019-3364-x.

27. Zhang H, Liu X, Xu C, Hu D, Li X, Li T, et al. Guiding Tuberculosis Control Through the Healthy China Initiative 2019-2030. China CDC Wkly. 2020;2(49):948-50. Epub 2021/10/02. doi: 10.46234/ccdcw2020.236. PubMed PMID: 34594807; PubMed Central PMCID: PMCPMC8393160.

28. Means AR, Kemp CG, Gwayi-Chore MC, Gimbel S, Soi C, Sherr K, et al. Evaluating and optimizing the consolidated framework for implementation research (CFIR) for use in low- and middle-income countries: a systematic review. Implement Sci. 2020;15(1):17. Epub 2020/03/14. doi: 10.1186/s13012-020-0977-0. PubMed PMID: 32164692; PubMed Central PMCID: PMCPMC7069199.

29. Ross J, Stevenson F, Lau R, Murray E. Factors that influence the implementation of e-health: a systematic review of systematic reviews (an update). Implement Sci. 2016;11(1):146. Epub 2016/10/27. doi: 10.1186/s13012-016-0510-7. PubMed PMID: 27782832; PubMed Central PMCID: PMCPMC5080780.

30. Lestari T, Graham S, van den Boogard C, Triasih R, Poespoprodjo JR, Ubra RR, et al. Bridging the knowledge-practice gap in tuberculosis contact management in a high-burden setting: a mixed-methods protocol for a multicenter health system strengthening study. Implement Sci. 2019;14(1):31. Epub 2019/03/21. doi: 10.1186/s13012-019-0870-x. PubMed PMID: 30890160; PubMed Central PMCID: PMCPMC6425655.

31. Narh-Bana SA, Kawonga M, Odopey SA, Bonsu F, Ibisomi L, Chirwa TF. Factors influencing the implementation of TB screening among PLHIV in selected HIV clinics in Ghana: a qualitative study. BMC Health Serv Res. 2022;22(1):898. Epub 2022/07/12. doi: 10.1186/s12913-022-08295-6. PubMed PMID: 35818070; PubMed Central PMCID: PMCPMC9272598.

32. Haldane V, Li BP, Ge S, Huang JZ, Huang H, Sadutshang L, et al. Exploring the translation process for multilingual implementation research studies: a collaborative autoethnography.

BMJ Glob Health. 2022;7(5). Epub 2022/06/01. doi: 10.1136/bmjgh-2022-008674. PubMed PMID: 35636804.

33. Sullivan-Bolyai S, Bova C, Harper D. Developing and refining interventions in persons with health disparities: the use of qualitative description. Nursing Outlook. 2005;53(3):127-33. doi: 10.1016/j.outlook.2005.03.005.

34. Sandelowski M. Whatever happened to qualitative description? Research in Nursing & Health. 2000;23(4):334-40. doi: 10.1002/1098-240X(200008)23:4<334::AID-NUR9>3.0.CO;2-G.

35. Sandelowski M. What's in a name? Qualitative description revisited. Research in Nursing & Health. 2010;33(1):77-84. doi: 10.1002/nur.20362.

36. Ritchie J, Lewis J, Lewis PoSPJ, Nicholls CM, Ormston R. Qualitative Research Practice: A Guide for Social Science Students and Researchers: SAGE; 2013 2013-11-01. 457 p.

37. Braun V, Clarke V. To saturate or not to saturate? Questioning data saturation as a useful concept for thematic analysis and sample-size rationales. Qualitative research in sport, exercise and health. 2021;13.

38. Haldane V, Zhang Z, Yin T, Zhang B, Li Y, Pan Q, et al. Strengthening rural tuberculosis health service delivery: A qualitative study with health workers in Tibet, China. Submitted to Plos Global Public Health. Forthcoming.

39. Long Q, Guo L, Jiang W, Huan S, Tang S. Ending tuberculosis in China: health system challenges. Lancet Public Health. 2021;6(12):e948-e53. Epub 2021/11/29. doi: 10.1016/S2468-2667(21)00203-6. PubMed PMID: 34838198.

40. Liu X, Blaschke T, Thomas B, De Geest S, Jiang S, Gao Y, et al. Usability of a Medication Event Reminder Monitor System (MERM) by Providers and Patients to Improve Adherence in

the Management of Tuberculosis. Int J Environ Res Public Health. 2017;14(10). Epub 2017/09/28. doi: 10.3390/ijerph14101115. PubMed PMID: 28946683; PubMed Central PMCID: PMCPMC5664616.

41. Zhang L, Wei G, Xu Z, Huang Q, Liu G. The Prevalence of Smartphones and WeChat Use Among Older Adults With Chronic Disease in a Western China. Comput Inform Nurs. 2020;39(1):42-7. Epub 2020/07/11. doi: 10.1097/CIN.0000000000000638. PubMed PMID: 32649363.

42. Morse RM, Myburgh H, Reubi D, Archey AE, Busakwe L, Garcia-Prats AJ, et al. Opportunities for Mobile App-Based Adherence Support for Children With Tuberculosis in South Africa. JMIR Mhealth Uhealth. 2020;8(11):e19154. Epub 2020/11/12. doi: 10.2196/19154. PubMed PMID: 33174850; PubMed Central PMCID: PMCPMC7688382.

43. Patel D, Berger CA, Kityamuwesi A, Ggita J, Kunihira Tinka L, Turimumahoro P, et al. Iterative Adaptation of a Tuberculosis Digital Medication Adherence Technology to Meet User Needs: Qualitative Study of Patients and Health Care Providers Using Human-Centered Design Methods. JMIR Form Res. 2020;4(12):e19270. Epub 2020/12/09. doi: 10.2196/19270. PubMed PMID: 33289494; PubMed Central PMCID: PMCPMC7755538.

44. Puchalski Ritchie LM, Kip EC, Mundeva H, van Lettow M, Makwakwa A, Straus SE, et al. Process evaluation of an implementation strategy to support uptake of a tuberculosis treatment adherence intervention to improve TB care and outcomes in Malawi. BMJ Open. 2021;11(7):e048499. Epub 2021/07/04. doi: 10.1136/bmjopen-2020-048499. PubMed PMID: 34215610; PubMed Central PMCID: PMCPMC8256754.

45. Atun R, Weil DE, Eang MT, Mwakyusa D. Health-system strengthening and tuberculosis control. Lancet. 2010;375(9732):2169-78. Epub 2010/05/22. doi: 10.1016/S0140-6736(10)60493-X. PubMed PMID: 20488514.

46. Stop TB Partnership. Key populations brief: Health care workers. 2016.

47. Xin H, Zhang H, Wang D, Zhang B, Cao X, Feng B, et al. The effect of WeChat-based training on improving the knowledge of tuberculosis management of rural doctors. J Clin Tuberc Other Mycobact Dis. 2021;25:100266. Epub 2021/08/31. doi: 10.1016/j.jctube.2021.100266. PubMed PMID: 34458591; PubMed Central PMCID: PMCPMC8379341.

48. Al-Shorbaji N. The World Health Assembly resolutions on eHealth: eHealth in support of universal health coverage. Methods Inf Med. 2013;52(6):463-6. Epub 2013/12/07. PubMed PMID: 24310395.

49. World Health Organization. Global strategy on digital health 2020-2025. Geneva: 2021.

50. The State Council of the People's Republic of China. China to further curb tuberculosis during 13th Five-Year Plan period (2016-2020) 2017 [cited 2023 3 January]. Available from: http://english.www.gov.cn/policies/latest_releases/2017/02/16/content_281475569328925.htm.

Supporting information

S1 File. Consolidated criteria for reporting qualitative studies (COREQ): 32-item checklist for each study

S1 File

Click here to access/download
Supporting Information
S1_File.pdf
