# Supplementary material for: A process evaluation of an eHealth intervention to strengthen the circle of tuberculosis care in Shigatse, Tibet, China
Source: PLOS Glob Public Health. 2025 Dec 23;5(12):e0005717. doi: 10.1371/journal.pgph.0005717 (PMC12725529; doi:10.1371/journal.pgph.0005717)
Supplement: Attachment — Submitted filename: Reply to reviewers.docx [file pgph.0005717.s005.docx]

Thank you for the opportunity to revise our manuscript, please find below our point-by-point reply to reviewers.

Reviewer #1: Your manuscript tackles an important and under-researched question: how multi-component digital adherence technologies can be implemented for TB control in a geographically remote, high-burden setting. Framing the study with the CFIR and reporting against COREQ demonstrates methodological intent, and the findings are immediately useful for TB programme managers. Nevertheless, several issues must be addressed to meet PLOS Global Public Health standards.

INTRODUCTION

1. Provide a brief rationale for choosing CFIR over alternative frameworks.

Thank you for this feedback, we have now added a rationale on line 113 which reads “The CFIR framework was used because it provides a comprehensive and flexible structure to identify multilevel factors, such as contextual, individual, and process-related elements, that influence the implementation of complex health interventions like eHealth for TB care in Shigatse.”

METHODS

1. Although it is mentioned that interviews ceased when no new codes emerged, it is suggested to describe how saturation was determined (operational criteria, evidence that the last 3-5 cases did not contribute new codes).

Thank you for the suggestion. We now say “Interviews were stopped when no new concepts emerged in the final three interviews for patients and providers respectively, indicating thematic saturation had been reached based on our operational criterion.”

2. Although it is mentioned that two analysts developed and refined the coding framework and resolved discrepancies through discussion. It is suggested to include examples of the code tree or a coded excerpt to illustrate the application of CFIR (and comply with item 25 of COREQ which appears as "N/A")

3. Justify sample size and provide numbers approached / refused (and comply with item 13 of COREQ which appears as "N/A")

We have updated item 13 in COREQ to say “All approached participants agreed to participate and no participants dropped out.” We have also added that our recruitment strategy was pragmatic and sensitive to the regional context. On Line 167 we now say “Purposeful and pragmatic recruitment was used that was sensitive to the resource constraints in the region (e.g., limited health workforce with little time, patients who traveled long distances or had other responsibilities), we also aimed for a balance of gender, age, health worker roles and location across four counties representing a mix of urban and rural locations.”

RESULTS
I recommend reporting the number of participants approached, refused, or excluded, and reasons where known.

We have added a sentence on line 208 explaining that “All approached participants agreed to be interviewed.”

DISCUSION

1. The manuscript briefly alludes to UTAUT framework to justify the relevance of digital adherence technologies, but in the Methods section and throughout the analysis — only uses CFIR. This isolated mention creates a "framework gap" that weakens the theoretical-methodological coherence.

Apologies, this was a typo from an earlier iteration and framing. We have removed this.

2. Consider referencing recent multicountry studies that have evaluated both barriers/facilitators and implementation outcomes of digital adherence technologies for TB—such as MERM, VDOT, and 99DOTS. Linking your findings to this broader literature will strengthen the discussion and highlight how your results compare with other implementation experiences.

Thank you, we have added a section that integrates with the broader literature beginning at line 533 “Future research should closely engage with decisionmakers to ensure implementation insights strengthen the delivery of high-quality TB care Recent multi-country studies have evaluated the implementation of digital adherence technologies, including Medication Event Reminder Monitors (MERM), Video Directly Observed Therapy (VDOT), and 99DOTS, highlighting both barriers and facilitators to their use. A scoping review using the RE-AIM framework found that while pillbox and video-based DATs had higher engagement rates, their reach was often constrained by cellphone access and technological challenges [51]. Similarly, the ASCENT trial conducted in the Philippines, South Africa, Tanzania, and Ukraine emphasized the importance of differentiated care models and found that effectiveness of digital adherence technologies varied by setting, with acceptability and feasibility shaped by local infrastructure and user needs [52].”

3. Acknowledge selection bias (participants recruited via the clinical trial) and possible social-desirability bias. It is suggested to also acknowledge Translation/interpretation bias, as cultural or technical nuances may be lost between Tibetan, Mandarin, and English.

Thank you for the suggestion, we have updated beginning at line 545 as follows, “A key limitation of the study is social desirability bias, where participants may have presented a more positively framed account of their engagement with the intervention. We attempted to limit this bias by reassuring participants that the study was not related to their TB care or work performance, and that all responses would be kept confidential. The impact of this bias was likely minimal, as participants were candid in sharing their perspectives, including negative ones. We also acknowledge selection bias, as participants were recruited through the clinical trial, which may have excluded individuals with differing experiences or levels of engagement. Additionally, translation and interpretation bias may have influenced the findings, as interviews conducted in Tibetan and Mandarin were translated into English, potentially losing cultural or technical nuances in the process, an idea which we have explored in our collaborative autoethnography on the translation process [32]. Although we tried to recruit a breadth of participants, vast travel distances and limited resources may have further constrained the diversity of perspectives, which could limit our interpretation of the implementation process.”

4. It is suggested to reflect on equity and transferability. What aspects are specific to the Tibetan context and which can be generalized to other remote, resource-limited settings?

Thank you for this suggestion, we have added a short paragraph at line 559: “While this study is grounded in the unique sociocultural and geographic context of Shigatse, several findings, such as the importance of tailoring digital tools to user needs, ensuring comprehensive health worker training, and adapting interventions to local infrastructure, are transferable to other remote, resource-limited settings. However, some cultural or health system aspects may be more specific to the Tibetan context or context of other rural settings in China and should be carefully considered when adapting similar interventions elsewhere. Reflecting on equity, future implementations must ensure that digital health tools are inclusive and accessible across age, education, and linguistic groups to avoid reinforcing existing disparities.”

5. The CFIR framework serves as a determinants model that systematically organizes barriers and facilitators affecting intervention implementation. However, CFIR alone does not define or assess Implementation Research Outcomes (IROs). To evaluate how well something was implemented, CFIR should be paired with outcome-focused frameworks, such as the widely-used Implementation Outcomes Framework (Proctor et al., 2011). The Discussion and Conclusion sections state that the intervention was “accepted” and “sustainable,” yet no IROs were measured to substantiate these claims. If the study’s explicit aim was solely to identify CFIR‐based determinants, this methodological boundary should be clearly stated in the Methods as a deliberate scope limitation. Consequently, the Discussion and Conclusions must remain aligned with the data actually collected, focusing on barriers and facilitators, and avoid drawing inferences about implementation outcomes that were not evaluated.

Thank you and we have updated throughout to make these boundaries clearer including noting in the methods sections that “Implementation research outcomes such as sustainability were not directly measured. As such, this evaluation focuses on understanding contextual and process-related factors influencing implementation, rather than assessing specific implementation outcomes.” Throughout the discussion and conclusion text we have also carefully edited to be clear that our insights may be useful for ongoing program delivery but that we did not specifically measure sustainability or similar IRO elements.

LANGUAGE AND PRESENTATION

The manuscript is generally intelligible but needs systematic copy-editing.

Thank you, we have now addressed these.

Typical issues include:

- Spelling mistakes “Directory Observed Therapy” : correct (Directly Observed Therapy)
- Repeated misspelling “Organizaton” : correct (Organization)
- Long sentences (>40 words) in Discussion—split for readability.
- Acronyms: present them in full the first time and maintain the same form ("DOTS" vs. "DOT")
- Maintain capitalization consistency (“e-Monitor” v. “e-monitor”)
- Verb tenses: in Results use consistent simple past ("participants reported" instead of alternating with present).
- Use of quotation marks: standardize single/double quotes
- "There are some incomplete sentences, for example: "Two reviewers (VH, ZZ) … discussed how the CFIR constructs.".

Reviewer #2: Overall, this is a study that demonstrates rigor and ethical responsibility; however, it has aspects that require further development. It is important to address how it was decided to work only on some CFIR constructs, as well as to review the UTAUT framework, which will be mentioned only at the end of the document.

Thank you for flagging this, it was a typo from an earlier approach and we have removed it.

The structure of the document is consistent with the requirements of the journal. The different sections reflect the expected content. However, the information available can be further leveraged to achieve solid results that can be useful for patients, individuals, and healthcare institutions.

The analysis plan requires an explanation of the process followed to arrive at the categories. In fact, no clear categories or subcategories that emerged from the process are addressed; only a more deductive than inductive analysis process is suggested. Some important elements are identified in the testimonies (see comments in the manuscript) that may lead to emerging findings of great value for the process.

Thank you, we have now included a coding tree that provides more elaboration on our findings.

Ethical considerations require clarity in the treatment of information from participants under 18 years of age, as the consent of parents or legal guardians is not sufficient.

We appreciate this comment and would like to clarify that while the RCT included participants under the age of 18, no participants under the age of 18 were interviewed for this process evaluation.

In the discussion, it is important to analyze the results in light of other studies and health policies that demonstrate the relevance of these results for the implementation of local, national, and global public policies, as well as the opportunity and need to develop new policies.

Based on the reviewers feedback, and that of reviewer 1, we have added more engagement with the literature including a new paragraph that elaborates on our findings in relation to other studies exploring digital adherence technologies.

In the attached manuscript you can see detailed observations that may be of interest to you in making adjustments to it.

Thank you for your comments to strengthen the manuscript.
