# Supplementary material for: A process evaluation of an eHealth intervention to strengthen the circle of tuberculosis care in Shigatse, Tibet, China
Source: PLOS Glob Public Health. 2025 Dec 23;5(12):e0005717. doi: 10.1371/journal.pgph.0005717 (PMC12725529; doi:10.1371/journal.pgph.0005717)
Supplement: Attachment — Submitted filename: Reply to reviewers_Revision 2.docx [file pgph.0005717.s006.docx]

Thank you for the opportunity to revise our manuscript, please find below our point-by-point reply to reviewers.

Reviewer #1: Thank you for submitting the revised version of your manuscript. I have analyzed the implemented changes and the response letter in detail, and I find that you have satisfactorily addressed all the suggestions made. The manuscript has improved considerably, especially in its methodological rigor and the contextualization of the findings within the existing literature. My following evaluation focuses on the implementation of these recommendations and offers a final critical perspective on a few aspects that could further strengthen the impact of your work.

1. A slight discrepancy has been identified regarding the description of data saturation. While the criterion has been rightly added to the Methods section, the COREQ checklist (S1 File) refers to the Results section for this point. Please ensure that the information in the checklist is harmonized to match the main text.

Thank you for bringing this discrepancy to our attention, we have updated the COREQ checklist.

2. The manuscript mentions that COVID-19 "shaped the inner setting" and will continue to affect the delivery of TB care. However, this idea is not further developed. Given that the recruitment period extended to the end of 2020, the pandemic was an unavoidable contextual factor. It would be enriching to add a sentence or two in the "Discussion" section reflecting on how the pandemic might have interacted with the intervention.

Thank you for highlighting this. We have added a note to the results section on the impact of COVID-19, starting at line 252 the section now reads “In addition, COVID-19 shaped the inner setting and continued to affect the delivery of TB care in the region. For example, during the COVID-19 pandemic, the home-visit DOT strategy was disrupted in some months due to isolation policies. However, patients receiving the intervention maintained patient management through remotely monitored medication boxes and WeChat-based communication.” We have also added a note into the discussion elaborating on how the intervention can support health services during disruptive events, culminating in the example of “For example, the COVID-19 pandemic disrupted TB health services in Tibet for some months due to public health and social measure policies, yet the intervention supported ongoing care delivery through remote monitoring during this time.” We would like to note that only one case of COVID-19 was reported in Tibet in 2020 and no further cases reported until 2022, so the COVID-19 situation in Tibet during data collection differed than other jurisdictions which had a larger burden of disease during 2020.

3. In the "Implementation Process" section, it is suggested that patients, family members, and community leaders can be "champions" to promote the intervention and TB awareness. This is a key implementation strategy. In the "Discussion" section, the importance of formalizing this role in future scale-up phases could be briefly mentioned. This would transform an interesting observation into a more solid and actionable programmatic recommendation.

Thank you for this suggestion, we have added some text on line 520 to elaborate a bit on this “Similarly, our work highlights an opportunity to work with village doctors to engage with patients and their families to promote community awareness of TB and support engagement with the intervention. Future research should leverage participatory approaches to explore how patients, their families, and community leaders could become champions of the intervention and TB awareness in the region.”

Reviewer #2: The authors have been clear in their responses to the reviewers' concerns and recommendations; however, while the reviewers' responses to comments are sometimes perceived as clear, they are not always as clear in the manuscript. I emphasize that the comments in the initial manuscript could contribute to improving the document. I mention one aspect as an example: it is clear that no minors under 18 years of age were interviewed, but for ethical reasons, it is understood that minors under 18 years of age did participate.

I recommend reviewing each of the answers and making sure that they are effectively reflected in the manuscript.

Thank you for this feedback, we have reviewed the manuscript against our prior replies to make sure it is clear. We have also directly updated the age limit and removed mention of participants under 18, since as we mentioned no one under 18 participated in the qualitative data collection that comprised this work.
