# Supplementary material for: A process evaluation of an eHealth intervention to strengthen the circle of tuberculosis care in Shigatse, Tibet, China
Source: PLOS Glob Public Health. 2025 Dec 23;5(12):e0005717. doi: 10.1371/journal.pgph.0005717 (PMC12725529; doi:10.1371/journal.pgph.0005717)
Supplement: Attachment — Submitted filename: Reply to reviewers_2.docx [file pgph.0005717.s007.docx]

Thank you for the opportunity to revise our manuscript, please find below our point-by-point reply to reviewers.

Reviewer #1: This revised manuscript represents an improvement over prior versions. The authors have addressed the majority of reviewers’ previous comments and aligned the paper with PLOS Global Public Health’s expectations. Minor editorial and conceptual adjustments are the only remaining issues before acceptance:

1. Although the COREQ checklist and S2 File indicate two coders, please add a line in the Methods describing how coding discrepancies were resolved.

Thank you for noting this, we have added a brief description to the methods at line 197 “After discussion of any discrepancies to reach agreement, the coding framework was applied to subsequent transcripts (S2 File).”

2. The Discussion section is overly detailed and extends beyond the recommended length for PLOS Global Public Health. Some paragraphs, particularly those describing user experiences with the e-Monitor and WeChat, reiterate similar points about accessibility and adaptation. These sections could be streamlined or synthesized to avoid redundancy, improve narrative flow, and highlight the most salient analytical insights.

Thank you for this feedback. We’ve re-written the discussion for clarity and brevity, while retaining the previously suggested elements from both reviewers (e.g., COVID-19 discussion, more engagement with the literature/other similar studies on implementation, etc).

3. Additionally, some minor writing errors that were previously flagged remain uncorrected, including inconsistent terminology (e.g., “Directly Observed Therapy” instead of “Director Observed Therapy”) and spelling inconsistencies (e.g., unify “e-Monitor” throughout the text).

We have updated the points raised by the reviewer.
